# Using 3D skeleton tracking for gait analysis in older adults compared to a kinect-based mobility analysis system

**DOI:** 10.1186/s12877-025-06763-2

**Published:** 2025-11-25

**Authors:** Kathrin Raeder, Nicole Strutz, Simone Kuntz, Sandra Strube-Lahmann, René Schwesig, Ursula Müller-Werdan, Nils Axel Lahmann

**Affiliations:** 1 Geriatrics Research Group, Charité - Universitätsmedizin Berlin (corporate member of Freie Universität Berlin and Humboldt-Universität zu Berlin), Berlin, Germany; 2 Department of Orthopedic and Trauma Surgery, Martin Luther University Halle- Wittenberg, Halle, Germany; 3 Munich Campus for Health and Engineering, Munich University of Applied Sciences HM, Munich, Germany

**Keywords:** Gait parameter, New algorithm, Older adults, Movement, App, Smartphone

## Abstract

**Background:**

Older persons are affected by falls and their associated consequences. This cohort is often unaware of their own risk of falling or misjudge it. Mobility and fall risk apps are therefore useful tools for recognizing fall risks and creating opportunities to reduce them. The aim of the study was to compare the agreement of a mobility analysis smartphone camera-based application (SCA) with an included advanced 3D skeleton tracking with the reference system Microsoft Kinect System^R^ (MKS).

**Methods:**

In a cross-sectional design, 186 data sets from 31 older adults were assessed at the same time with both systems depending on two gait modes (comfort and fast).

**Results:**

For the comfort mode, the ICC values ranged from 0.74 (gait speed; 95% CI: 0, 0.93) to 0.85 (cadence; 95% CI: 0.62, 0.93). The fast mode showed a slightly lower agreement between both systems. ICC values moved from 0.66 (gait speed; 95% CI: 0, 0.90) to 0.81 (cadence; 95% CI: 0.46, 0.91).

**Conclusion:**

With the exception of gait speed, both systems demonstrated good agreement (ICC > 0.75) in measuring spatiotemporal gait parameters. These findings suggest that the SCA has the potential to serve as a reliable and practical instrument in the context of clinical gait analysis.

## Background

One of the most common events worldwide that endanger or impair the health of people aged 60 or older or lead to death are falls. Every year, more than 37 million falls occur that are serious enough to require medical treatment [1]. Knowledge of fall risk factors such as age, frailty, fear of falling and polypharmacy has been documented for many years [2–5]and is taken into account in the professional care context through professional assessment, established fall risk evaluations, and measures to reduce the risk of falling [6]. However, often older individuals do not seek the assessments and expertise of healthcare professionals regarding fall risk [7]. Older people often underestimate their own risk of falling and assume that they are not at risk of falling [8–10], thus seeing no reason to seek help from therapeutic or nursing professionals. This can lead to serious health consequences. One option to reliably assess their own risk of falling with minimal effort is to use fall risk and mobility smartphone applications that can be used by non-experts [11, 12]. In the course of digitalization, there are now a few mobility apps available. One such mobility app is a German mobility analysis smartphone camera-based application (SCA).

The SCA is designed to detect and reduce fall risks. Input parameters to compute the fall risk score included video analysis of each person’s gait through an artificial intelligence–based algorithm and a standardized questionnaire that assesses fall risk factors. The SCA is not only based on the measurement of functional ability but also incorporates intrinsic factors and utilizes 3D skeleton tracking for gait analysis. The SCA was further developed with an algorithm based on 3D skeleton tracking to determine the risk of falling more reliably.

The objective of the study was to verify the agreement of a modular algorithm of a mobility SCA, which employs 3D skeleton tracking technology compared with an established and frequently used system (MKS).

## Methods

### Apparatus

The Motognosis Lab Software (Motognosis GmbH, Berlin, Germany) using a Microsoft Kinect System (MKS) and the modular algorithm of a mobility app (SCA) with 3D skeleton were used to conduct the gait analysis. MKS was chosen as an internationally recognized reference standard [13].

MKS: The Motognosis Lab Software using a Microsoft Kinect System is an assessment of motor behaviour. For this purpose, a 3D/RGB camera (Microsoft Azure Kinect V4) was used to capture depth silhouettes of individuals using visual perception computing. Based on 32 anatomical orientation points in 3D, a movement was calculated using Azure Kinect Body Tracking SDK (v1.1.0). The Microsoft Azure Kinect V4 scans the environment in three dimensions using infrared light analysis and is able to accurately measure shapes and movements in the space in front of it. The camera tracks body parts with millimetre accuracy, without the need for markers or special clothing. For each motor task, such as walking, specific kinematic outcome parameters were developed to describe motor behaviour. These are used to validate recordings or in exploratory evaluation of movement patterns.

Mobility app: The mobility smartphone application on a smartphone with a camera was used in order to evaluate gait and fall risk. The scientific basis of the app is based on a modular algorithm that includes a video tester, a skeletal estimator (skeletal estimator 2D, skeletal estimator 3D, skeletal optimization 3D), and an analysis of mobility parameters. The aim is to offer users a comprehensive gait analysis and provides users with a customized catalogue of measures aimed at fall prevention, tailored to the specific needs of older adults. These recommendations are designed to support and maintain physical mobility. The testing is restricted solely to the recording of mobility parameters using camera-based systems. The collection of additional parameters relevant to fall analysis, such as those obtained via questionnaires, was not included.

SCA: The modular algorithm skeleton estimator is a crucial component, as the validity of both the mobility parameters and the overall analysis depends significantly on its spatial and temporal precision [14]. SCA was installed on an Android-based Smartphone.

**Fig. 1:**
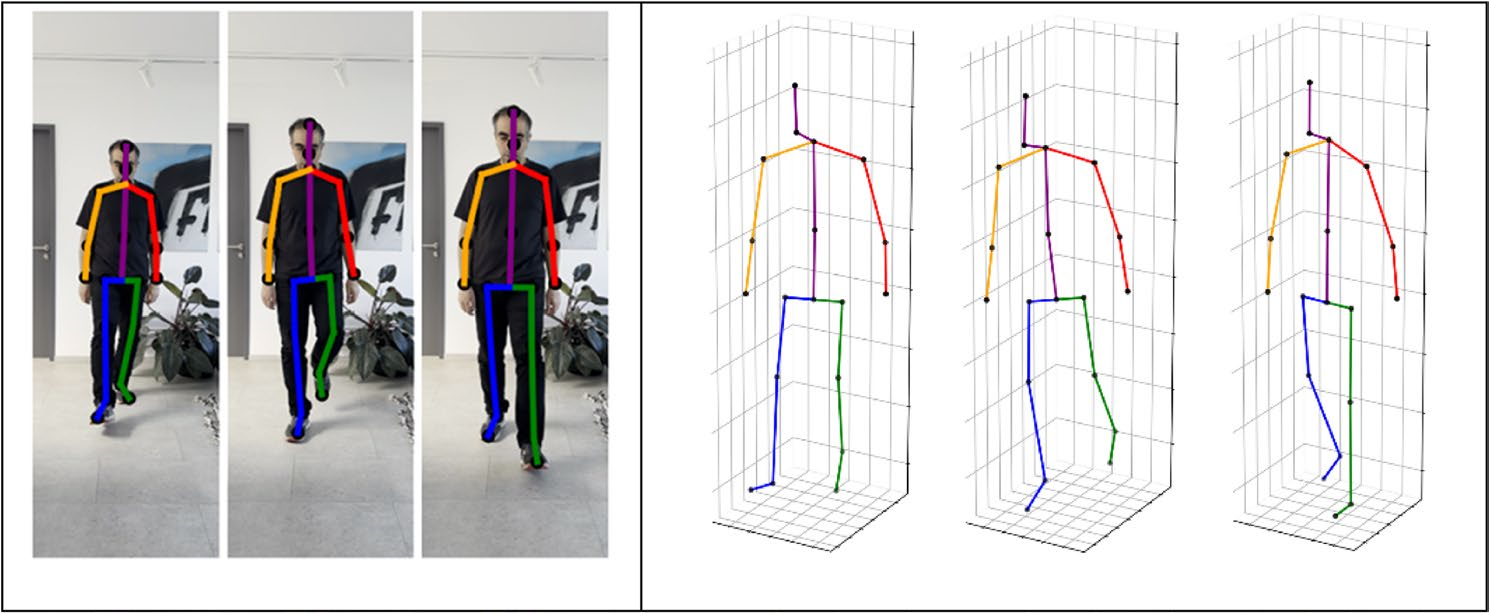
Two-dimensional (left) and corresponding three-dimensional (right) pose estimations using a 19-keypoint skeleton model from the gait analysis algorithm

To assess the gait, the app’s artificial intelligence (AI) analyses a short video recorded on a smartphone. The assessment process consists of four main steps. First, a module tests the video quality to ensure it meets the necessary standards for accurate skeleton estimation. The skeletal estimator then analyses the video frame by frame, identifying joint coordinates and estimating the skeleton in three-dimensional space with millimetre precision (Fig. 1). This involves neural networks specialized in image processing, which first extract a 2D skeletal structure before transforming it into 3D. Subsequently, the app calculates gait parameters based on the estimated joint data, including the average gait speed, step length, and step time as well as the cadence for the entire video. Finally, the analysis module generates a personalized report based on the computed gait parameters.

The Mobility Analysis app allows for the precise recording of human movements and their conversion into de-tailed, digital 3D models.

The skeletal estimator is central to the modular algorithm, as the accuracy of the mobility parameters and analysis is highly dependent on its spatiotemporal precision. Neural network-based 3D human pose (skeletal) estimators have been developed and trained using extensive datasets, consisting of millions of images paired with skeletal sensor data as ground-truth labels [15]. The algorithm is capable of delivering highly precise and valid gait parameters, provided it processes anatomically correct skeletal data. For further technical details on the algorithm, refer to [14].

SCA integrates multiple algorithmic modules into an end-to-end pipeline that has been optimized for unsupervised, smartphone-based gait assessment in older adults. The key advancements are:

1. **Hybrid 2D–3D skeleton estimation with anatomical fitting.**

The pipeline begins with a customized 2D skeleton tracker (CPM/HRNet variant) and a 3D tracker (VNect/GASTNet variant) trained and fine-tuned for gait-related lower-limb landmarks. These outputs are then processed by our proprietary kinematic skeleton fitter, which enforces anatomically consistent bone lengths and joint angles using a Ceres-based optimization. This ensures that the reconstructed 3D skeleton is biomechanically plausible, even from monocular video.

2. **Patented gait-event detection.**

A patented algorithm identifies “honest extrema” in ankle–pelvis distance trajectories, enabling robust extraction of step events from noisy monocular skeleton data. This step-event detection method is unique to our system and distinguishes it from research prototypes relying on unsmoothed 2D trajectories.

3. **Robustness modules for real-world clinical use.**

Unlike PoseNet, OpenPose, or MediaPipe, which assume controlled conditions, the SCA includes dedicated pre-processing modules: camera-movement detection (optical flow, accelerometer), person detection (Faster R-CNN), and re-identification with user confirmation (Torch-ReID). These safeguard against moving cameras, multi-person interference, or occlusions—failure modes common in unsupervised care environments.

4. **Clinical validation and regulatory robustness.**

The novelty lies not only in technical integration but in clinically validated performance. The SCA achieved ICC values ≥ 0.97 for gait speed, cadence, and step time against GAITRite, demonstrating reliability comparable to gold-standard systems [14]. This level of validation, particularly in older adults, goes beyond open-source frameworks that have not been tested in clinical populations.

To make these distinctions explicit, we have prepared the following comparison (Table 1):

**Table 1:** Comparison between SCA, PoseNet, openpose and MediaPipe in Ters of feature/criteria

Feature/Criterion	SCA (Smartphone Camera Application)	PoseNet	OpenPose	MediaPipe
**2D Pose Estimation**	CPM/HRNet variant (optimized for gait in older adults)	MobileNet/ResNet	Part Affinity Fields	BlazePose
**3D Skeleton Estimation**	VNect/GASTNet + kinematic fitter (anatomical)	✗	Limited via external models	BlazePose 3D (no anatomical fitting)
**Anatomical Consistency**	Enforced bone/joint constraints	✗	✗	Partial smoothing
**Step Event Detection**	Patented extrema detection	✗	✗	✗
**Robustness Modules**	Camera QC, person detection, re-ID	✗	✗	✗
**Clinical Validation**	ICC ≥ 0.97 vs. GAITRite	None	None	Limited
**Target Use Case**	clinical fall-risk analysis	Fitness apps	Research	Fitness/wellness
**Platform**	Mobile (Android, iOS), CE-certified	Mobile/Web	Desktop/Server	Mobile

The system uses open scientific backbones for the initial pose estimation steps but extends them with proprietary modules designed to achieve anatomically consistent 3D skeletons, robust gait-event detection, and clinically reliable output:

- **2D pose estimation**: Based on variants of the Convolutional Pose Machine (CPM) and HRNet, which provide 2D joint coordinates per frame.
- **3D lifting**: Implemented through VNect- and GASTNet-like networks that project 2D joint sequences into 3D coordinates.

#### Proprietary extensions (novelty)

1. **Kinematic Skeleton Fitter** – This module is unique to our system. It combines 2D and 3D joint detections with subject height to fit an anatomically plausible skeleton. It uses a least-squares optimization (Ceres solver) with four terms: inverse kinematics, projection back to 2D, temporal smoothness, and depth regularization. The result is a 3D skeleton with stable trajectories and biomechanically valid bone lengths, which is critical for clinical gait assessment.
2. **Patented step-event detection algorithm** – After skeleton fitting, step cycles are extracted not by raw extrema but by a clustering approach that identifies “honest extrema” in ankle–pelvis distances. This method is patented and ensures robust detection of gait events even under noisy monocular conditions, allowing accurate computation of cadence, step length, and step time.
3. **Video quality and robustness modules** – Unlike generic pose-estimation frameworks, our system integrates dedicated pre-analysis checks:

- **Camera movement detection** via optical flow and sequence rejection if instability is detected.
- **Person detection** with Faster R-CNN to isolate the walking subject.
- **Person re-identification** (Torch-ReID) with user confirmation to ensure the correct individual is consistently tracked.

Together, these extensions transform general-purpose 2D/3D pose estimation into a clinically robust gait analysis pipeline that is safe for unsupervised use in older adults.

## Difference from existing frameworks

- PoseNet, OpenPose, and MediaPipe generate raw 2D keypoints (and in some cases approximate 3D uplift) but do not enforce anatomical validity, perform step-event detection, or integrate video quality assurance.
- None of these frameworks has demonstrated validation against GAITRite in clinical populations.
- By contrast, our SCA, with its proprietary extensions, achieves ICC ≥ 0.97 for gait speed, cadence, and step time in validation studies [14].

The description of the SCA system’s algorithmic pipeline used the following training dataset, architecture or backbone of the AI model and the way the 3D estimation is computed from 2D frames.

- **Training datasets (what we use)**: For 2D pose, SCA employs HRNet backbones with pre-trained weights released via MMPose on COCO-WholeBody and MPII; these weights are referenced in our 2D module specification and are used without modification in production builds. For 3D pose uplifting, SCA uses a pre-trained GASTNet (video-based) model as provided; our documentation specifies the model integration and pre-trained checkpoint use. Clinical validation was performed on the Charité and HPI reference datasets against GAITRite ground truth, as documented in the verification report.
- **Model backbones (what we run)**: The 2D stage supports CPM and HRNet; HRNet is our default in production (top-down: detector/segmentation → HRNet keypoints). The 3D stage implements GASTNet for spatiotemporal 2D→3D uplifting and includes an implementation of VNect; these appear in our prior methodological description of the SCA modules. On top, we run a proprietary kinematic skeleton fitter (least-squares with Ceres) that enforces anthropometric consistency and temporal smoothness.
- **Computation from 2D to 3D (how it works)**: After 2D keypoints are extracted, frames are validated (must contain a usable 2D skeleton) and normalized by centring image coordinates and mapping width to [− 1, 1]; batching is used for efficiency. The 3D uplift yields joint coordinates in a pelvis-centred system. We then run the kinematic fitter, which jointly aligns (i) the 3D uplift, (ii) the 2D reprojection, and (iii) temporal priors. The energy has four terms—inverse-kinematics consistency, 2D projection error (also recovering camera-relative position), temporal smoothness, and depth regularization—as detailed in our published SCA module description. Bone lengths are estimated on high-quality frames, scaled to subject height, and used as constraints during fitting. Outputs include camera-relative joint positions and joint angle trajectories for gait analysis.

**Fig. 2:**
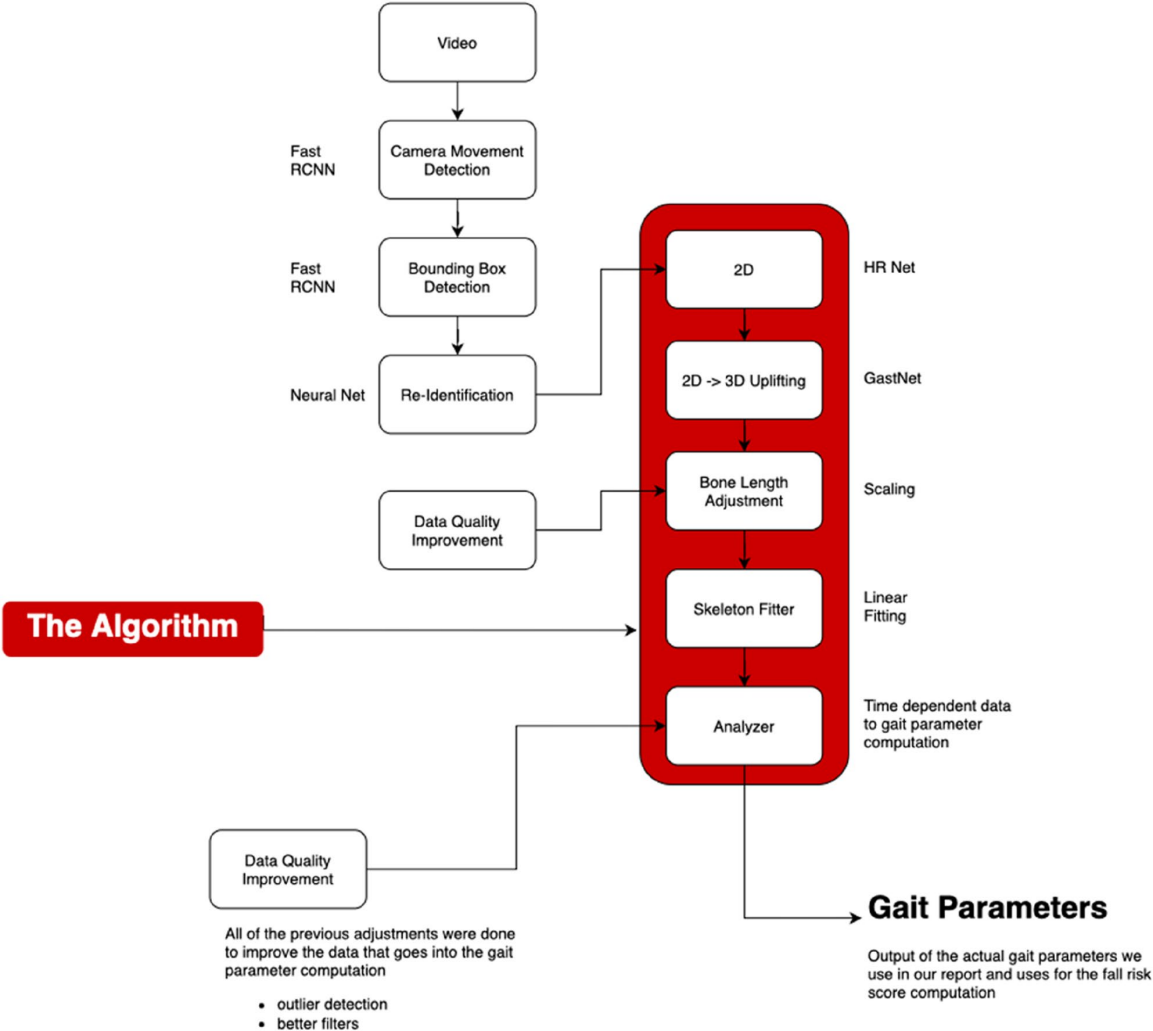
Pipeline video to gait parameter extraction

**Fig. 3:**
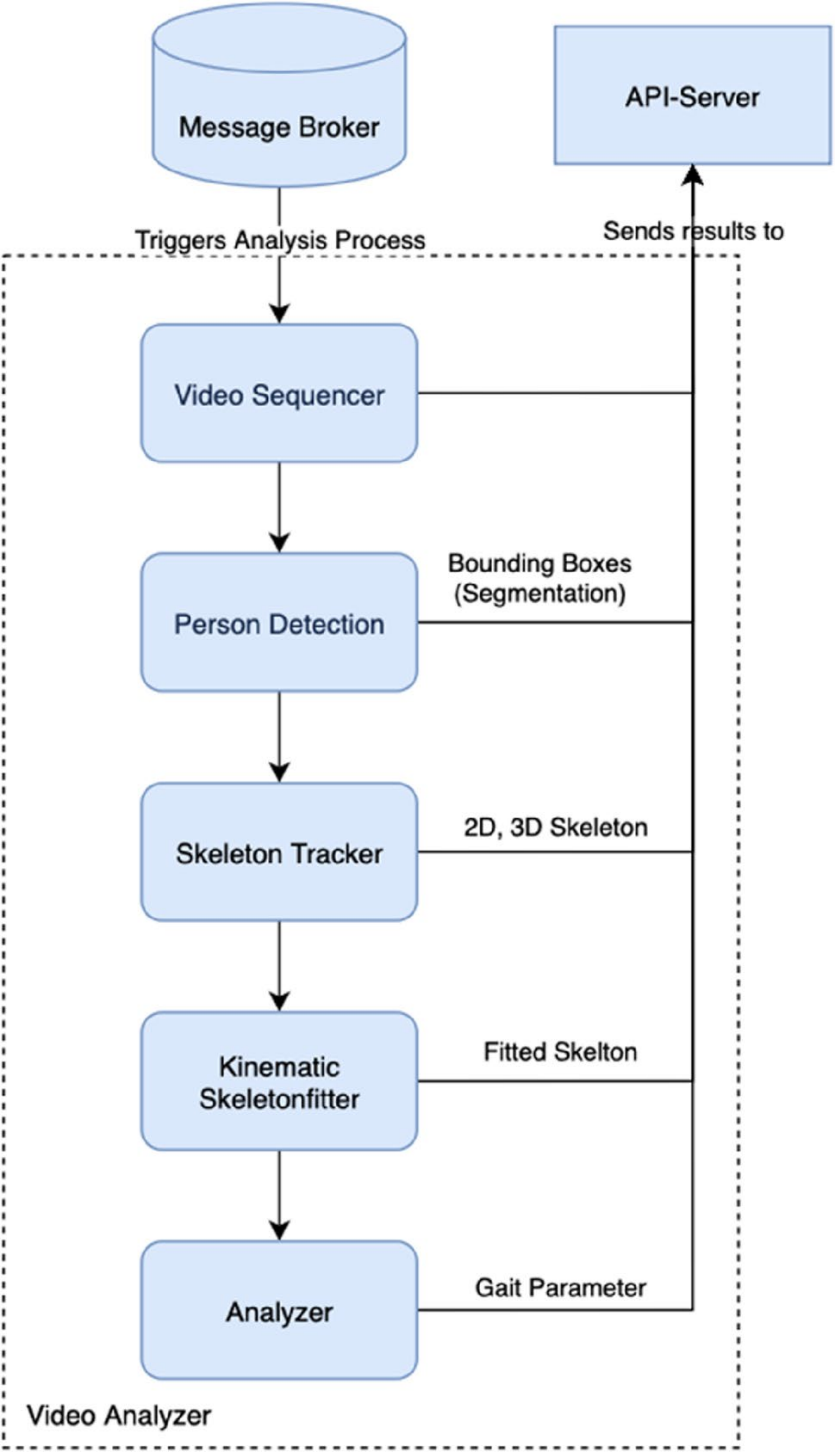
Video Analyser Engine

The SCA is built as a modular video analysis chain demonstrating the pipeline from video to gait parameter extraction (Fig. 2) and video analyser engine (Fig. 3).

**Fig. 4:**
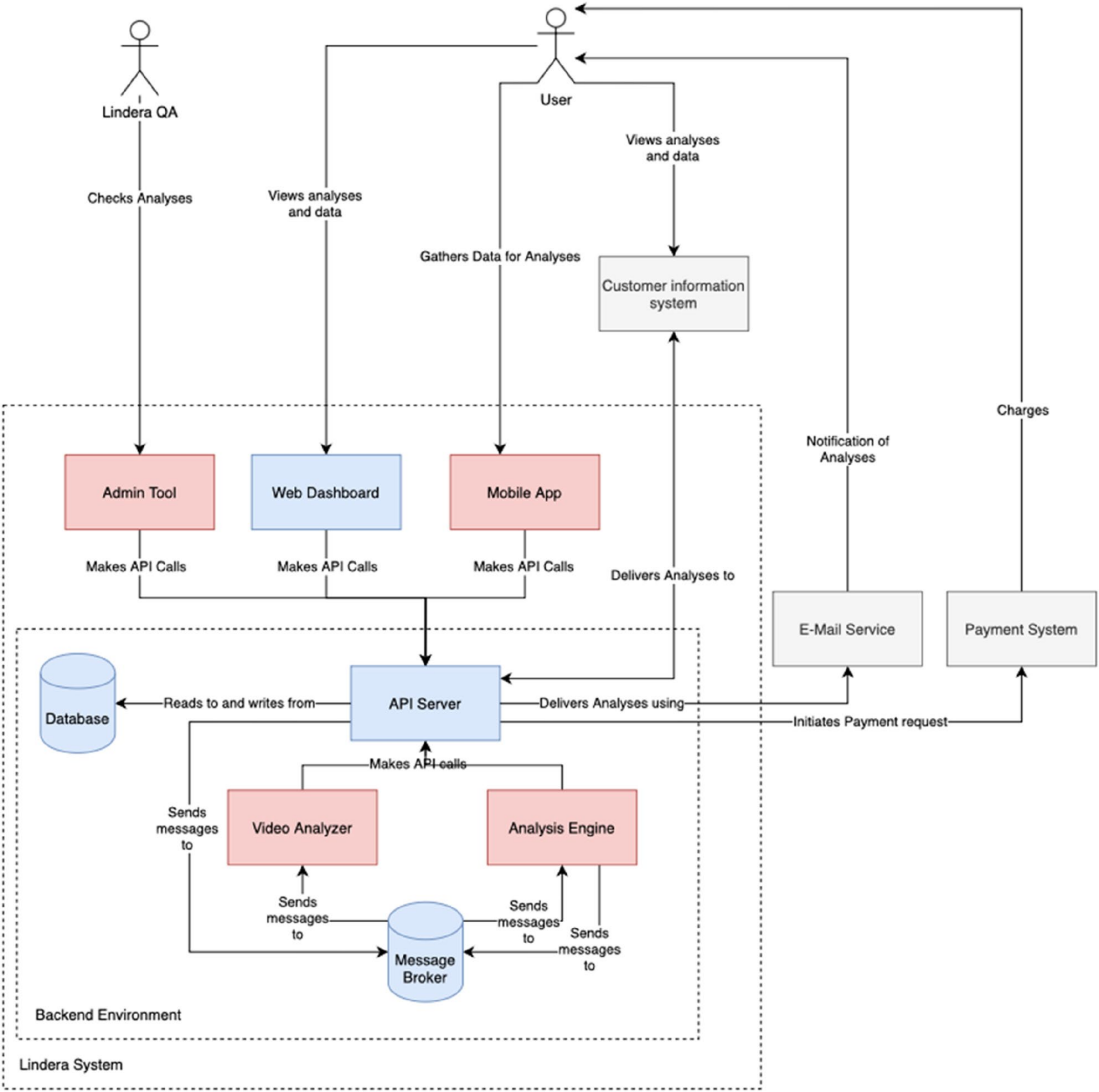
Application system architecture

To enhance traceability of the system architecture, below is an overview of the application system architecture (Fig. 4).

For this study the gait analysis of the mobility analysis camera-based app (SCA) was used alongside the reference gait analysis system (MKS).

### Procedure

Study participants were recruited through two primary channels: (1) the Geriatrics Research Group database comprising older adults who had previously provided informed consent to be contacted for research participation (2), the dissemination of a study poster within a geriatric hospital setting. Inclusion criteria stipulated an age threshold of 65 years or older, as well as the capacity for ambulation and the ability to rise from and return to a seated position. The use of walking aids, such as wheeled walkers or crutches, was permitted. Individuals with severe cognitive, sensory or motor disorders and those with a legal representative were excluded.

**Fig. 5:**
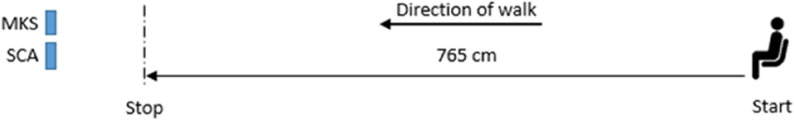
Experimental Setup, graphic chart

**Fig. 6:**
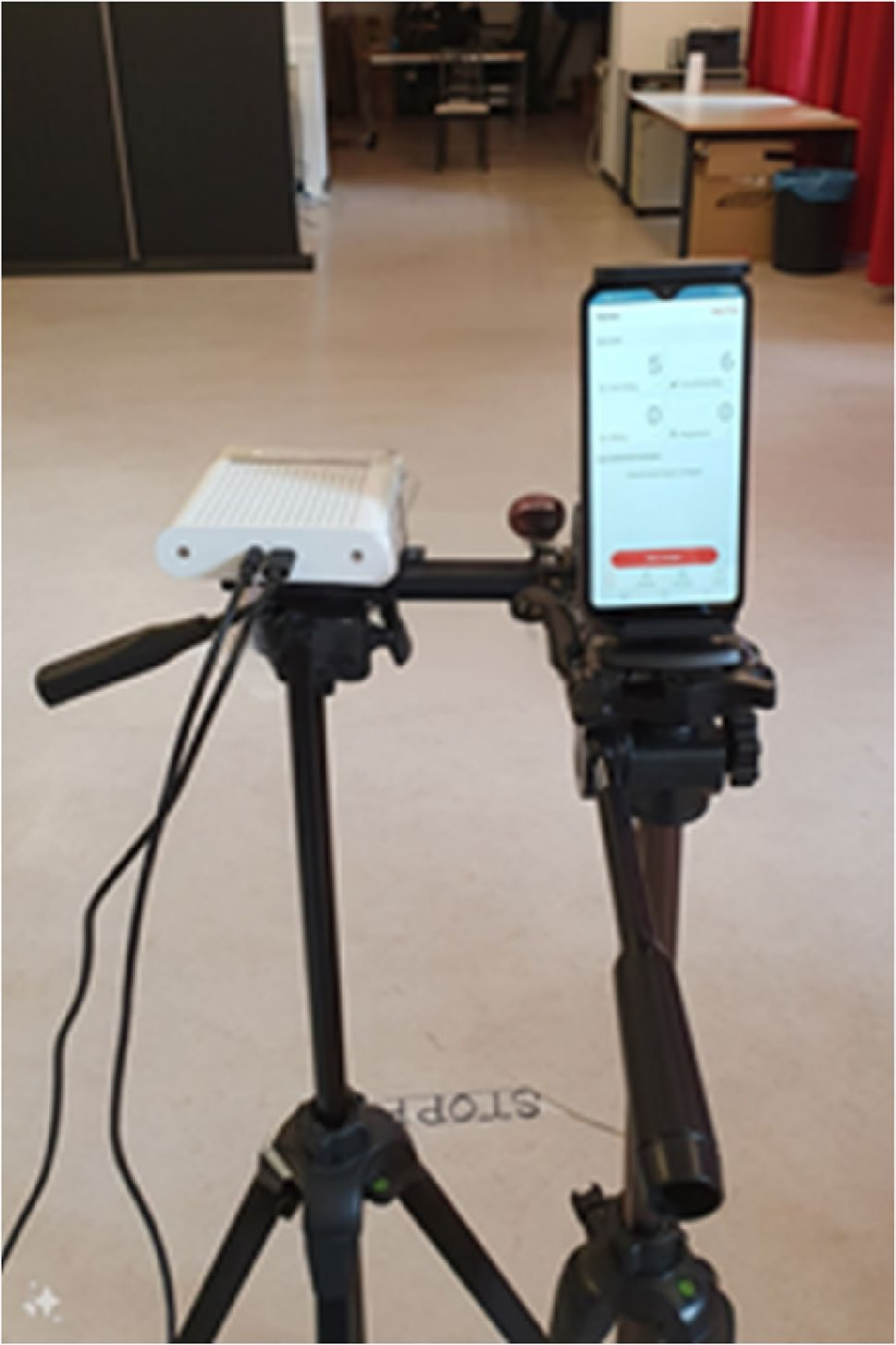
Experimental Setup, MKS on the left and SCA on the right side

The gait analysis was carried out at the laboratory of the Geriatrics Research Group in Berlin. The study was approved by the local ethics committee at Charité—Universitätsmedizin Berlin and conducted according to the Declaration of Helsinki in its currently applicable version. All participants gave written informed consent. The participants completed a self-reporting questionnaire covering sociodemographic data such as age and sex. Height, weight and leg length were measured by the study staff. Subsequently, each participant performed six walks under two speed conditions: comfort mode and fast mode. The first three walks were at a self-selected usual speed over a distance of 7.65 m, while the subsequent three walks were to be completed at a faster speed at the same distance, instructed as a velocity faster than their usual walking speed (Figs. 5 and 6). The length of the Lab allowed a running distance of 7.65 m in order to guarantee the longest possible recording length.

Prior to each walking trail, both the MKS and the SCA systems,, which are aligned frontally with the older adults, were activated to enable the simultaneous analysis of participants’ gait patterns (Fig. 6). Video recording of both the MKS and SCA systems was initiated as participants entered the designated walking area. Following each walking trial, study personnel conducted a verification process to ensure the accuracy of the recorded measurements. The total duration of each study visit was approximately one hour per participant.

### Gait parameters and statistical analysis

The following spatiotemporal gait parameters were measured and descriptively reported (mean ± standard deviation (SD)): gait speed [m/s], cadence [Hz], stride length [cm] and step time [s]. Anthropometric (e.g., leg length, weight) and sociodemographic data (e.g., age, sex) were collected. The data of MKS and SCA were time stamped and merged. The statistical analysis of the collected data was conducted using SPSS version 27.0 for Windows (IBM, Amonk, NY, USA).

The Bland-Altman Plot (B-A Plots) was used as a graphical representation method for the comparison of two measurement methods (MKS vs. SCA).

The Intraclass Correlation Coefficient (ICC, 2,k – two-way random, absolute agreement, average measurement) was used to calculate the agreement between the two measurement methods. The Pearson correlation (r) measures the linear relationship between two variables and can provide an indirect indication of agreement. Values close to 1 indicate strong positive linear correlation, suggesting similarity in measurements. However, Pearson correlation measures association rather than agreement and can be high despite systematic differences (bias). Therefore, it is insufficient alone to evaluate interchangeability or accuracy. Nonetheless, due to its sensitivity to linear relationships, robustness to measurement units, and ease of interpretation, it serves as a useful initial metric. Complementary methods such as the intraclass correlation coefficient (ICC) and Bland-Altman analysis are recommended for comprehensive assessment [16, 17]. The ICC (2,k) is a crucial metric for assessing the reliability of measurements, particularly in studies involving multiple observers or measurement instruments. An ICC (2,k) value close to 1 indicates high agreement between measurements, suggesting high reliability. An ICC (2,k) value close to 0 indicates low agreement between measurements, suggesting low reliability. The ICC values are classified as follows: values below 0.5 are considered poor, values between 0.5 and 0.75 are considered moderate, values between 0.75 and 0.9 are considered good, and values above 0.9 are considered very good [18]. 95% confidence intervals (95% CI) were reported for ICC and r values.

## Results

### Sample

The baseline characteristics of the study population, including age, height, weight and leg length (left, right) are presented in Table 1. A total of 31 participants were included in the study (female *n* = 17, 55%). The mean age of the older adult studied was 77.7 years (SD: 5.4 years) (Table 2).

**Table 2:** Baseline data reported as mean ± SD

	total (*n* = 31)	female (*n* = 17)	male (*n* = 14)
age (years)	77.7 (5.4)	78.2 (6.0)	77.0 (4.7)
height (cm)	170 (9.0)	165 (7.1)	175 (8.1)
weight (kg)	74.6 (13.3)	67.4 (7.8)	83.3 (13.5)
leg length left (cm)	102 (6.5)	100 (6.0)	104 (6.8)
leg length right (cm)	102 (6.3)	100 (5.7)	104 (6.7)

### Agreement of two measurements

A total of 185 walks were conducted (*n* = 31 participants). Of these 95 walks were performed in comfort mode (CM), while 90 walks were conducted in fast mode (FM), based on the participants’ walking abilities. Due to a small number of missing data the number of walks available for each variable ranged from 79 to 93.

**Fig. 7:**
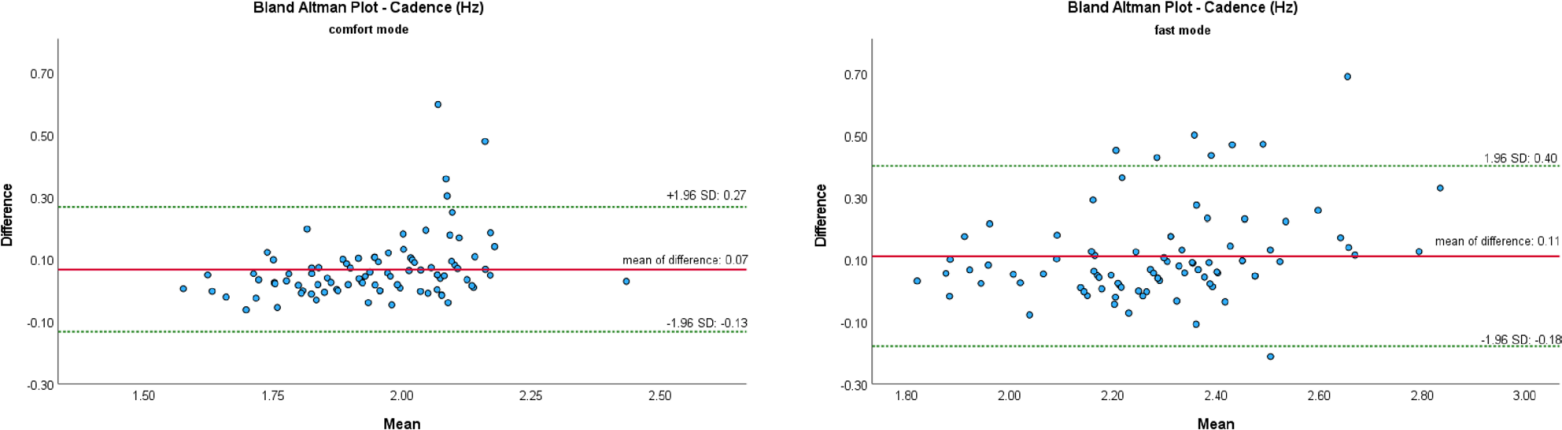
Bland Altman Plots of MKS and SCA for cadence depending on gait mode: comfort vs. fast

**Fig. 8:**
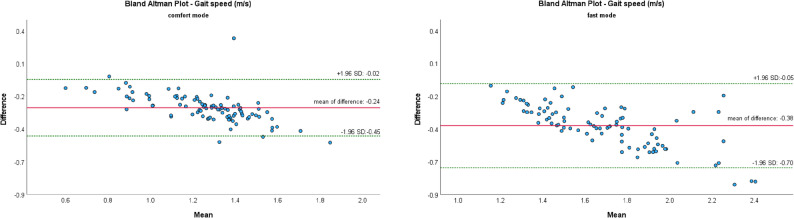
Bland Altman Plots of MKS and SCA for gait speed depending on gait mode: comfort vs. fast

**Fig. 9:**
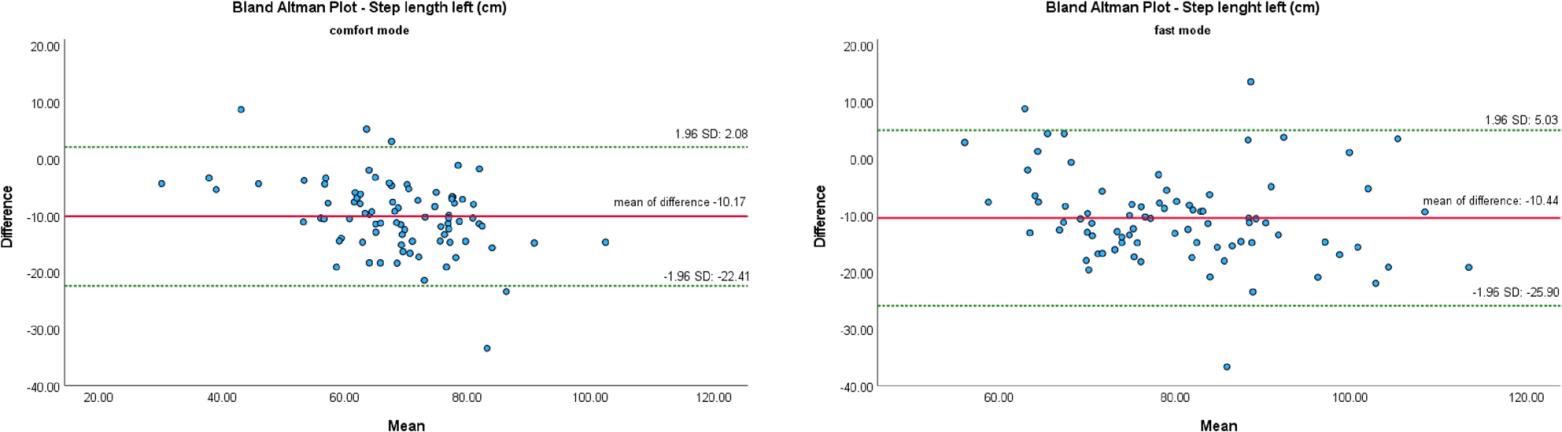
Bland Altman Plots of MKS and SCA for step length left depending on gait mode: comfort vs. fast

**Fig. 10:**
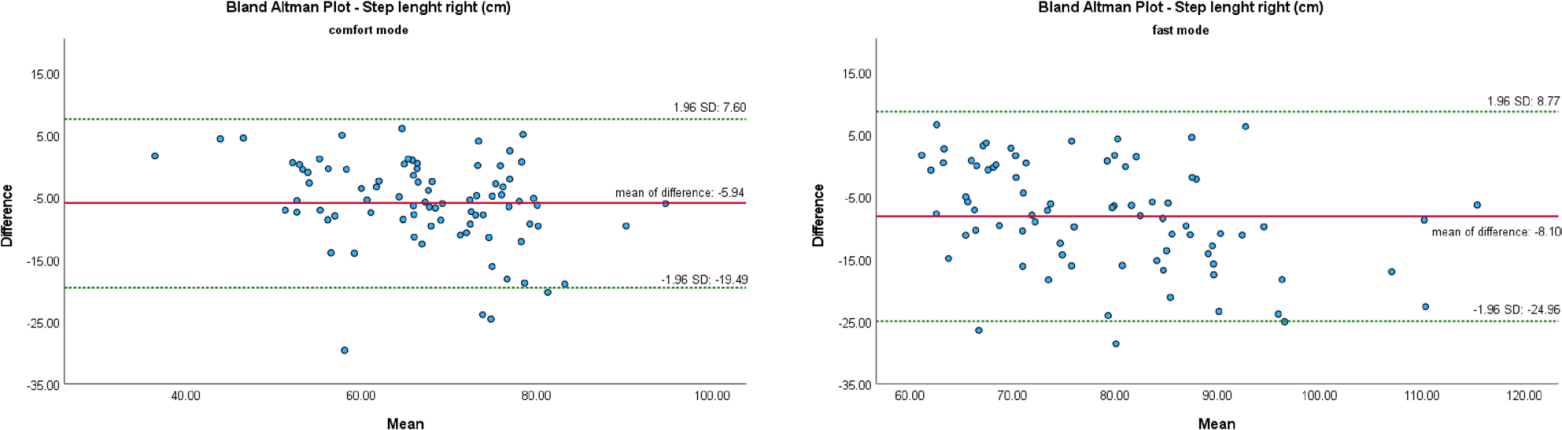
Bland Altman Plots of MKS and SCA for step length right depending on gait mode: comfort vs. fast

**Fig. 11:**
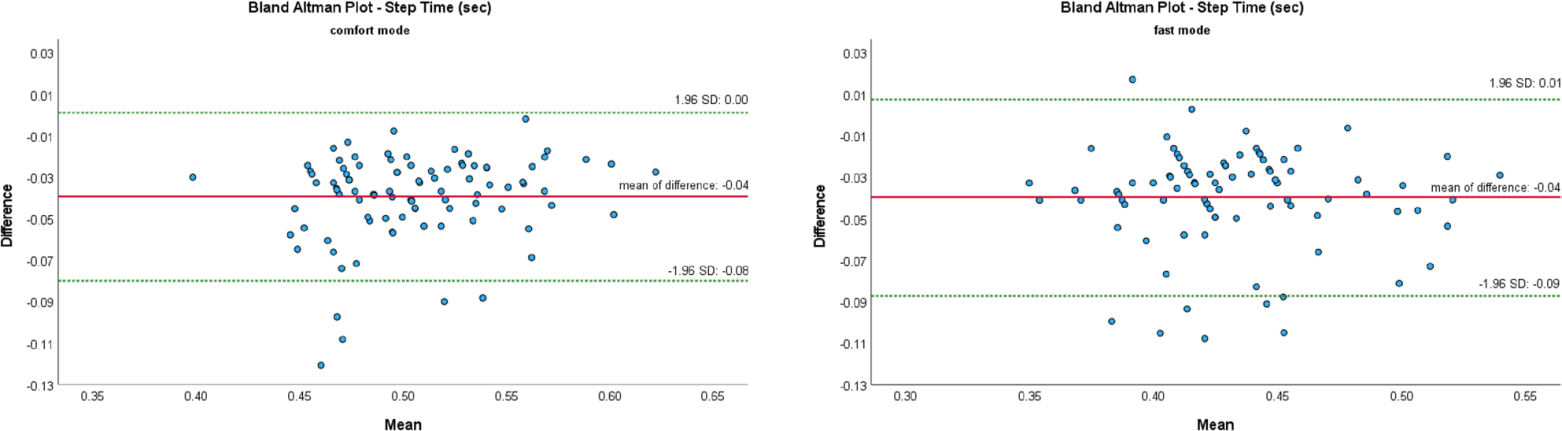
Bland Altman Plots of MKS and SCA for step time depending on gait mode: comfort vs. fast

Figures 7, 8, 9, 10 and 11 show Bland-Altman plots of the five gait parameters (gait speed [m/s], cadence [Hz], step length left & right [cm] and step time [s]) for the two speed conditions: comfort mode and fast mode. The limits of agreement (LOA) were represented by the green lines and indicates the upper and lower limits of the acceptable differences, which are defined as ± 1.96 standard deviations. The red line represents the mean value of the differences.

The majority of data pairs exhibited values within the predetermined LOA. Eight outliers were identified in the parameters of cadence (fast mode) and step time (fast mode). In comfort mode, five outliers were observed in the variables of step time, step length left, and gait speed.

The Pearson correlation values (r) of the measured spatiotemporal gait parameters for the Microsoft Kinect System (MKS) and the smartphone camera-based application (SCA) ranged between 0.82 and 0.93 in the CM and between 0.79 and 0.93 in the FM (Table 3).

**Table 3:** Comparison of MKS and SCA depending on several modes (comfort vs. fast) - descriptive data (mean ± standard deviation (SD)) and testing of agreement based on ICC and pearson correlation (r) analysis

Gait mode	parameter	MKS (mean ± SD)	SCA (mean ± SD)	*r*	95% CI	ICC	95% CI
comfort	**cadence** (Hz)	1.98 ± 0.18	1.91 ± 0.15	0.82	0.74, 0.88	0.85	0.62, 0.93
	**gait speed** (m/s)	1.14 ± 0.20	1.38 ± 0.27	0.93	0.89, 0.95	0.74	0, 0.93
	**step length**,** left** (cm)	63.6 ± 10.8	73.7 ± 13.2	0.88	0.83, 0.92	0.78	0, 0.93
	**step length**,** right** (cm)	64.4 ± 9.97	70.4 ± 12.0	0.82	0.73, 0.88	0.83	0.38, 0.93
	**step time** (s)	0.49 ± 0.04	0.53 ± 0.04	0.89	0.83, 0.92	0.76	0, 0.93
fast	**cadence** (Hz)	2.34 ± 0.24	2.23 ± 0.20	0.79	0.69, 0.86	0.81	0.46, 0.91
	**gait speed** (m/s)	1.50 ± 0.24	1.87 ± 0.36	0.93	0.89, 0.95	0.66	0, 0.90
	**step length**,** left** (cm)	75.1 ± 12.1	85.5 ± 13.7	0.82	0.73, 0.88	0.76	0, 0.92
	**step length**,** right** (cm)	75.0 ± 11.3	83.1 ± 14.4	0.80	0.71, 0.87	0.79	0.22, 0.92
	**step time** (s)	0.41 ± 0.04	0.45 ± 0.04	0.82	0.74, 0.88	0.72	0, 0.91

The ICC values demonstrated a good agreement between MKS and SCA for the following parameters: cadence (0.85), step length left (0.78) and right (0.83) and step time (0.76) in comfort mode, and cadence (0.81), step length left (0.76) and right (0.79) in fast mode. The parameters gait speed (CM 0.74, FM 0.66) and step time (FM 0.72) demonstrated moderate agreement.

## Discussion

The purpose of the present study was to examine the reliability of the 3D skeleton tracking algorithm of an application for gait analysis in older adults. The results demonstrated a good to moderate agreement between the SCA and MKS. The SCA seems to be a practical solution for gait analysis compared to laboratory-based systems such as MKS.

The smartphone camera-based application (SCA) demonstrated consistently high Pearson correlations (*r* = 0.79–0.93) with the Microsoft Kinect System (MKS), indicating a strong linear relationship in the assessment of spatiotemporal gait parameters. Importantly, the corresponding ICC values suggest good to moderate agreement for most clinically relevant parameters—particularly cadence, step length (left, right), and step time—across both comfort and fast mode conditions. The strong correlations for gait outcomes such as gait speed (*r* = 0.93) and cadence (*r* = 0.82–0.79), together with favorable ICC values (up to 0.85 for cadence, 0.78–0.83 for step length, 0.76–0.81 for step time), highlight the potential of the SCA as a reliable tool for gait assessment in clinical practice. While some limitations remain, for example the only moderate agreement observed for gait speed during fast walking (ICC = 0.66), the overall findings indicate that the SCA provides sufficiently robust measurements for practical application, thereby supporting its integration as an accessible and low-cost alternative to established motion analysis systems.

The relevance of the complex issue of falls is recognized not only in professional fields but also in society at large, including its economic implications. Awareness of existing fall risk factors remains crucial. For instance, the Apple watch (Apple Corporation, Cupertino, USA) detects fall events and Apple claims that the iPhone (ios 15) can infer fall risk factors based on the user’s gait [19]. However, the high cost of such devices limits their accessibility. In contrast, the SCA, combined with a widely available smartphone, offers an affordable and practical solution for gait and fall risk assessments. Furthermore, the SCA provides a more comprehensive assessment by including personal and environmental risk factors beyond gait patterns, giving it a distinct advantage.

Shafi et al. [20] evaluated the smartphone application G&B that also validated gait parameters. The G&B app showed a similarly high reliability of the gait parameter gait speed (ICC: 0.93, 95% CI: 0.89, 0.96) against clinical outcomes (Berg Balance Scale, Timed Up & Go Test, Functional Reach Test) compared to the SCA (comfort mode) gait speed reliability (ICC: 0.74, 95% CI:−0.16, 0.93). The G&B app primarily focuses on balance tasks similar to well-known analogue mobility tests, without assessing critical fall risk factors such as gender, fear of falling, or polypharmacy. While the G&B app is intended as a supplement to clinical assessments, it is not designed for independent use by non-professionals. In contrast, the smartphone camera-based application (SCA) app provides a broader range of assessments, empowering non-professionals to assess their fall risk independently and reliably.

In comparison with the study by Steinert et al. [21]which employed 2D skeleton tracking, the present study, which employed 3D skeleton tracking, demonstrated significantly higher levels of agreement in various gait parameters in both gait modes (comfort mode and fast mode). It is important to note that in the study of Steinert et al. the two systems (MKS and SCA) were not tested against each other, but in each case with the GAITRite system. The MKS demonstrated a high level of agreement (CM ICC 2,k: minimum 0.90; FM ICC 2,k: minimum 0.81), while the SCA exhibited a poor level of agreement (CM ICC 2,k: minimum 0.09; FM ICC 2,k: minimum − 0.08) [21]. In a further study by Azhand et al. [14], an updated SCA with 3D skeleton tracking system was tested once more with GAITRite system. This demonstrated enhanced values of 0.96 (ICC 2, k) [14].

Considering the age of study participants with a minimum of 72.3 and a maximum of 82.7 years among men and a minimum of 72.2 and a maximum of 84.2 years among women it becomes evident that the younger-old and the oldest-old groups were not included in the study. One might infer that the study results could differ for these two unrepresented age groups. However a study byAzhand et al. (2021) using the reference system GAITRite reported similar outcomes, with participant ages ranging from 65 to 91 years [14]. For future studies, reliance on convenience sampling should be avoided; instead, targeted stratification by age groups should be employed to enable valid additional validations.

As these gait parameters are highly relevant for assessing mobility and are critical measures of functional ability [22], this significant improvement highlights the progression of the app.

The Bland-Altman plot revealed several outliers that fell outside the previously defined limits of agreement. These suggest that the agreement between the two camera systems may be limited in specific measurement scenarios. Potential sources of these variations, including differing device positioning, device movements during recording, variable lighting conditions, and individual movements of the subjects, were ruled out through simultaneous recording. However, it remains uncertain whether the use of an alternative smartphone could reduce these outliers, a factor that should be taken into account in further system development.

### Limitations

It is assumed that the participants do not correspond to the population average of over 70-year-olds in terms of gait and body position characteristics. Based on voluntary participation, the possibility of a selection bias cannot be discounted. The experimental design was documented in a protocol, but a potential bias in the data due to the involvement of different researchers cannot be excluded. The generalizability is limited due to the lack of external validation or independent dataset. Further research is needed to explain the differing results when comparing the SCA with MKS and GAITRite system.

## Conclusion

While the MKS remains a reliable and validated tool for determining gait parameters, its utility is largely confined to research and clinical settings due to its infrastructure and cost requirements. The SCA, on the other hand, offers an accessible solution, enabling individuals to reliably assess their fall risk using 3D skeleton tracking without needing specialized equipment or expertise. The findings of this study underscore the accuracy of the further developed algorithm of SCA, as measured compared with the established and frequently used MKS. These results highlight the potential of the SCA as a reliable, accessible, and comprehensive tool for gait analysis and fall risk assessment in older adults.

## Data Availability

The datasets generated and/or analyzed during the current study are available from the author Simone Kuntz (simone.kuntz@charite.de) on reasonable request.

## Abbreviations

B-A Plot: Bland-Altman Plot
CI: Confidence interval
LOA: Limits of agreement
MKS: Microsoft Kinect System^R^
SCA: Smartphone camera-based application
SD: Standard deviation
